# The Atypical Setting: The Challenging Care of Migrants

**DOI:** 10.1192/j.eurpsy.2026.11640

**Published:** 2026-07-31

**Authors:** S. Bellissima, C. Lo Cicero, G. Pellegrino

**Affiliations:** 1 Medicina della Migrazione e delle Emergenze Sanitarie, ASP 3 Catania; 2 Istituto Cirino La Rosa, Catania, Italy

## Abstract

**Introduction:**

Psychological care for migrants poses significant challenges. Many arrive with severe psychopathological conditions such as PTSD, complex PTSD, depression, and psychosis, often stemming from trauma related to war, violence, persecution, or extreme socio-economic hardship. Upon first contact, migrants frequently show profound distrust toward healthcare professionals, associating therapeutic settings with past hostile experiences. Many are unfamiliar with psychological concepts, often confusing therapy with medical examinations. This distrust is heightened when mental health referrals come without clear explanation, making patients feel unprepared or threatened by potentially judgmental or legal situations, like child welfare interventions. Additionally, cultural differences deeply influence diagnosis, as definitions of mental illness vary widely. Western diagnostic models like the DSM-5-TR may pathologize behaviors seen as normal or adaptive in other cultures. Furthermore, psychological research predominantly reflects WEIRD (Western, Educated, Industrialized, Rich, Democratic) perspectives, limiting its relevance and risking cultural misinterpretations in migrant care.

**Objectives:**

create favorable conditions for communication and integration; to improve therapeutic compliance; to promote mental health and well-being through flexible, culturally-sensitive approaches that deviate from conventional methods, based on deeper knowledge of the patient’s background.

**Methods:**

Therapeutic settings are adapted to cultural differences, such as using WhatsApp consultations with Nigerian shamans to engage reluctant patients. Hypnosis, combined with virtual reality, offers immersive, culturally transcendent experiences and was presented at EPA Congress 2025 (EPV1038). A triadic setting includes a cultural mediator fluent in language, culture, healthcare, and therapy dynamics, fostering trust between mediator and psychotherapist. To reduce cultural bias in assessments, “culture-free” psychometric tools like Raven’s Progressive Matrices for intelligence and the Rorschach test for personality are employed.

**Results:**

The implementation of these interventions yielded the following outcomes: reduction in anxiety and recovery time; decreased reliance on psychotropic medication; strengthening of the ego and enhancement of self-esteem; improved acceptance of therapeutic engagement; increased levels of social and cultural integration.

**Conclusions:**

This work is an everyday challenge, still working on critical points and learning new things.

**Disclosure of Interest:**

None Declared

